# In silico DNA methylation analysis identifies potential prognostic biomarkers in type 2 papillary renal cell carcinoma

**DOI:** 10.1002/cam4.2402

**Published:** 2019-07-30

**Authors:** Man Yang, Ryan A. Hlady, Dan Zhou, Thai H. Ho, Keith D. Robertson

**Affiliations:** ^1^ Department of Molecular Pharmacology and Experimental Therapeutics Mayo Clinic Rochester Minnesota; ^2^ Department of Nephrology Shanghai General Hospital, Shanghai Jiao Tong University Shanghai China; ^3^ Division of Hematology and Medical Oncology Mayo Clinic Scottsdale Arizona

**Keywords:** biomarkers, epigenetics, epigenomics, gene copy number

## Abstract

There are currently no effective treatments for advanced‐stage papillary renal cell carcinoma (PRCC). The goal of this study is to define potential DNA methylation‐based markers and treatment targets for advanced‐stage type 2 PRCC. Progressive DNA methylation changes and copy number variation (CNV) from localized to advanced‐stage type 2 PRCC are analyzed by using methylation data generated by TCGA's kidney renal papillary cell carcinoma (TCGA‐KIRP, 450k array) project. Survival analyses are performed for the identified biomarkers and genes with CNV. In addition, expression of the corresponding genes is investigated by RNA‐seq analysis. Progressive methylation changes in several CpGs from localized to advanced‐stage type 2 PRCC are observed. Four CpGs (cg00489401, cg27649239, cg20555674, and cg07196505) in particular are identified as markers for differentiating between localized and advanced‐stage type 2 PRCC. Copy number analysis reveals that copy gain of *PTK7* mostly occurs in advanced‐stage type 2 PRCC. Both the four CpG methylation changes and *PTK7* copy number gain are associated with patient survival. RNA‐seq analysis demonstrates that *PTK7* copy gain leads to higher *PTK7* expression relative to tumors without copy number gain. Moreover, *PTK7* is significantly upregulated from localized to advanced‐stage type 2 PRCC and is linked to cancer cell invasion. In conclusion, DNA methylation markers that differentiate between localized and advanced‐stage type 2 PRCC may serve as useful markers for disease staging or outcome, while *PTK7* copy gain represents a potential treatment target for advanced‐stage type 2 PRCC. Stepwise methylation changes and copy number gain also associate with disease stage in PRCC patients.

## Graphical abstract

In the present study, we identified four DNA methylation markers that distinguished between localized and advanced‐stage type 2 papillary renal cell carcinoma (PRCC) with excellent sensitivity and specificity. Moreover, these four DNA methylation markers may be useful for predicting patient outcome since they were significantly associated with patient survival. Furthermore, copy number analysis revealed that gain of PTK7 occurred most frequently in advanced‐stage tumors, predicted poor survival, and was associated with upregulated PTK7 expression in advanced‐stage disease. This finding strongly suggests that PTK7 represents a potential therapeutic target in advanced‐stage type 2 PRCC.

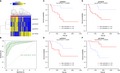

## 1 INTRODUCTION

Papillary renal cell carcinoma (PRCC) is the second most common type of renal cell carcinoma (RCC), accounting for 15%‐20% of all RCC cases.^1^ According to histological characterization, PRCC is divided into type 1 and type 2.^1^ Type 2 PRCC tends to present with more advanced stage, more frequent vascular invasion or distant metastasis, and consequently significantly worse clinical outcome.^2^, ^3^ There are currently no effective treatments for advanced‐stage PRCC.

Alterations in DNA methylation have been observed in PRCC and are associated with tumor stage and clinical prognosis.^4^ *RASSF1A* is frequently hypermethylated, with less prevalent hypermethylation of *APC*, *CDH1*, and *GSTP1*.^5^ Moreover, *CDH1* methylation is linked to pathologic stage and patient survival.^5^ The Cancer Genome Atlas (TCGA) Research Network reports distinct DNA methylation patterns between type 1 and type 2 PRCC.^1^ Furthermore, type 2 is further stratified into three subgroups based on multiplatform omics analysis (copy number, DNA methylation, gene expression, miRNA expression, and protein expression). Each of the three type 2 PRCC subgroups display differential methylation patterns, one linked to a CpG island methylator phenotype (CIMP) phenotype and the other two each showing distinct DNA methylation clusters (cluster 1 & 2) through genome‐wide profiling based on the Illumina Infinium HumanMethylation450k array (450k, TCGA‐KIRP project).

While PRCC comprises 15%‐20% of all RCCs, the overwhelming majority of analyses are focused on the most frequent subtype, clear cell RCC, leaving a gap in knowledge for PRCC etiology, especially at the epigenetic level. To address this issue, we examine 450k data from TCGA's KIRP dataset with a specific focus on identifying DNA methylation alterations and copy number variations (CNVs) that can be linked specifically to advanced‐stage PRCC. Our analysis reveals that key regulatory regions (CpG islands, promoters, and enhancers) are dominated by hypermethylation events, with relatively fewer DNA hypomethylation events distinguishing advanced‐stage PRCC. Due to the reversibility of DNA methylation, coupled with the limited treatment options available for PRCC, we sought to identify novel therapeutic targets and epigenetic markers for PRCC. To that end, we identify a subset of CpGs (cg00489401, cg27649239, cg20555674, and cg07196505) and a CNV (*PTK7* amplification) that differentiate localized from advanced‐stage type 2 PRCC, demonstrating that both genetic and epigenetic features likely contribute to disease progression.

## 2 MATERIALS AND METHODS

### 2.1 The Cancer Genome Atlas (TCGA) data

TCGA‐KIRP 450k array data were downloaded from theGenomic Data Commons (GDC) (https://gdc-portal.nci.nih.gov/legacy-archive/search/f). Ten patients were reclassified based on review by a group of expert pathologists, as described in the TCGA‐KIRP publication.^1^ We utilize the reclassified type 1 and type 2 data in this analysis: eight patients (TCGA‐AL‐7173, TCGA‐B1‐A47M, TCGA‐B9‐7268, TCGA‐BQ‐7060, TCGA‐BQ‐7062, TCGA‐EV‐5901, TCGA‐HE‐A5NI, and TCGA‐PJ‐A5Z8) are reclassified as type 1 while two patients (TCGA‐A4‐8098 and TCGA‐J7‐8537) are reclassified as type 2.

### 2.2 450k array data analysis

Raw data from the 450k array are normalized by the Subset‐quantile Within Array Normalization (SWAN) using the R package “RnBeads” (version 1.6.1).^6^ After filtering out CpGs on allosomes (leaving 473 921 CpGs) and adjusting for age and gender, differentially methylated probes (DMP) are defined as having a change in methylation (delta beta)> |0.1| and a false discovery rate (FDR) adjusted *P* value < 0.05.

### 2.3 Ingenuity pathway analysis

Pathways are identified and analyzed using Ingenuity Pathway Analysis (IPA, http://www.qiagen.com/ingenuity). The significance of genes in each pathway is determined using a right‐tailed Fisher's exact test. The top five significant pathways in each comparison are selected for presentation.

### 2.4 Marker analysis

For classification with the nearest shrunken centroid method, the R package “pamr” (version 1.48.0) is used.^7^ After 10‐fold cross validation and setting the shrinkage threshold to 5.404, a list of CpGs capable of discriminating between localized and advanced‐stage type 2 PRCC is generated. CpGs that progressively gain or lose methylation from localized to advanced‐stage type 2 PRCC are defined using the R package “pROC” (version 1.14.0) to perform receiver operating characteristic (ROC) analysis for a multivariable logistic regression model.^8^

### 2.5 Copy number analysis

The R package “ChAMP” (version 1.8.2) is used to analyze copy number aberrations using IDAT data from the 450K array.^9^ Data are linked to genes using the R package “CNTools” (version 1.26.0).^10^ Genes are considered significantly altered if the segment mean value is > |0.2| and more than 20% of samples are affected with FDR < 0.05.

### 2.6 Survival analysis

Kaplan‐Meier survival curves are calculated and plotted in R using the “survplot” function of the “rms” package.^11^(version 4.5‐0).^9^ The log rank test is used to compare the survival differences between the groups; *P* < 0.05 is regarded as significant.

### 2.7 RNA‐seq expression analysis

RNA‐seq raw gene counts for TCGA‐KIRP are downloaded from GEO accession number GSM1536837 (tumor) and GSM1697009 (adjacent‐normal). Differential gene expression analysis is performed using the R package “EdgeR” (version 3.12.1).^12^ Genes with absolute logarithmic fold change (logFC) > 0.5 and FDR < 0.05 were considered differentially expressed.

## 3 RESULTS

### 3.1 Progressively gained or lost DNA methylation from localized to advanced‐stage type 2 PRCC

**Figure 1:**
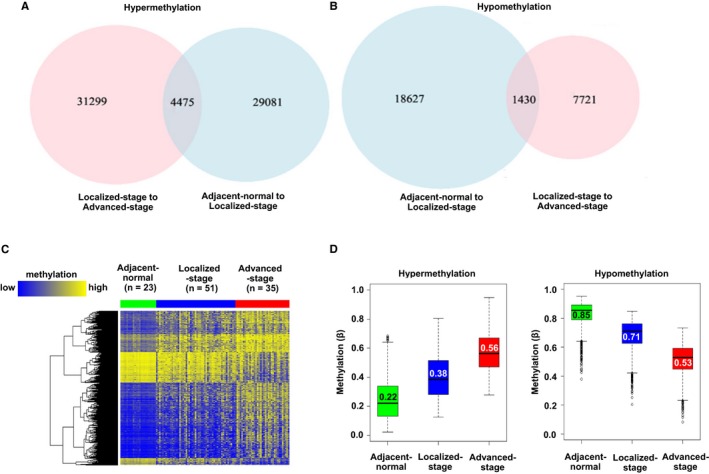
Progressive methylation changes from localized to advanced‐stage type 2 papillary renal cell carcinoma (PRCC). (A‐B) Venn diagrams summarizing the number of CpGs showing progressive (A) hypermethylation or (B) hypomethylation. Overlapping regions of the Venn diagram denote progressive changes. (C) Heatmap of CpGs with progressive methylation changes. Green: adjacent‐normal tissue; blue: localized‐stage type 2 PRCC samples; red: advanced‐stage type 2 PRCC samples. A color bar is shown, with low methylation in blue, and high methylation in yellow. (D) Boxplots showing the methylation levels of CpGs with progressive methylation changes in adjacent‐normal, localized, and advanced‐stage type 2 PRCC samples, respectively. The lower and upper whiskers encompass the first and fourth quartiles, respectively, with outliers demarcated by open circles. The second (bottom) and third (top) quartiles are contained within the colored boxes separated by the black median bar. The median value is shown within the box

Stages I‐II are defined as localized, and stages III‐IV are considered as advanced PRCC, which provided 51 cases of localized‐stage type 2 PRCC, 35 cases of advanced‐stage type 2 PRCC, and 23 cases of adjacent‐normal tissue from the TCGA‐KIRP dataset. The clinicopathologic characteristics of type 2 PRCC patients enrolled in this study are summarized in Table S1. Our results show that 4475 CpGs progressively gain methylation while 1430 CpGs progressively lose methylation (Figure 1A,B) when comparing advanced to localized stages. These alterations are also stepwise in nature from adjacent‐normal to localized to advanced stage (Figure 1C,D).

### 3.2 Feature‐based analysis of CpGs with progressive DNA methylation changes from localized to advanced‐stage type 2 PRCC

**Figure 2:**
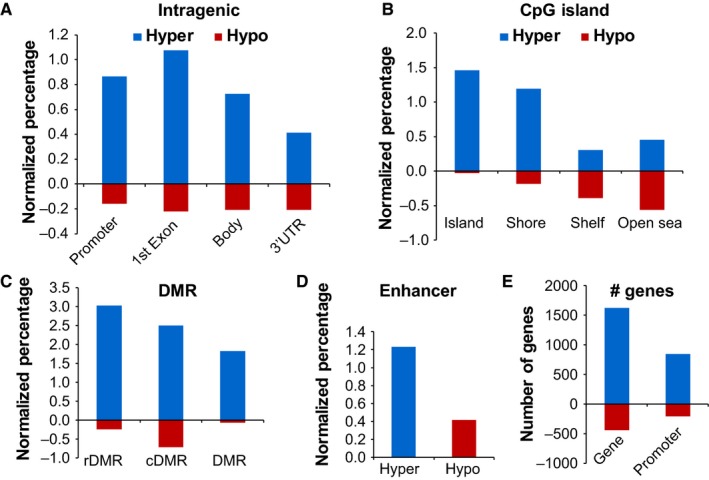
Genomic feature annotation of CpGs showing progressive methylation changes from localized to advanced‐stage type 2 PRCC. Intragenic‐ (A) and CpG island‐ (B) based features for CpGs showing progressive hypermethylation or hypomethylation linked to progression. DMR (C) and enhancer (D) analysis of CpGs showing progressive methylation changes. In (A‐D), the number of changes is normalized to the total number of CpGs within each given feature on the 450k array to allow for comparison between features. (E) The number of genes and promoters linked to CpGs with progressive hypermethylation or hypomethylation. In all bar charts, hypermethylation is colored in blue, and hypomethylation in red

From localized to advanced‐stage, the majority of progressive hypermethylation changes occur at promoters (including TSS1500, TSS200, and 5′‐UTR) and first exon regions, while progressive hypomethylation changes are evenly distributed across intragenic regions (Figure 2A). Furthermore, progressive gains of methylation are mostly localized at or adjacent to CpG islands, while progressive loss events occur primarily outside of CpG islands (Figure 2B). Differentially methylated region (DMR) analysis shows that progressive hypermethylation is distributed at reprogramming‐specific differentially methylated regions (rDMR), cancer‐specific differentially methylated regions (cDMR), or other DMRs genome‐wide, while progressive hypomethylation is primarily localized to cDMRs (Figure 2C).^13^ More enhancers (1.2%) progressively gain methylation than lose methylation (0.4%, Figure 2D). While 1620 genes (from 848 gene promoter regions) progressively gain methylation, 442 genes (from 206 gene promoter regions) progressively lose DNA methylation (Figure 2E). Taken together, these findings indicate that DNA hypermethylation, particularly at gene regulatory elements, is the dominant change occurring during the progression of PRCC from early to advanced stage.

### 3.3 Functional analysis of genes undergoing progressive DNA methylation changes from localized to advanced‐stage type 2 PRCC

To investigate the potential functional consequences of DNA methylation changes that occur between localized and advanced‐stages, pathway analysis was performed for genes linked to progressively hyper‐ or hypomethylated CpGs located within gene promoters. Finally, 848 genes corresponding to 1419 CpGs with progressive promoter hypermethylation, and 206 genes corresponding to 258 CpGs with progressive promoter hypermethylation were further analyzed. Genes showing progressive promoter hypermethylation are associated with adrenergic signaling, G protein signaling, and Gamma aminobutyric acid (GABA) receptor signaling, while genes with progressive promoter hypomethylation are associated with immune response, cytokine production, and the inflammasome pathway (Tables S2 and S3). Diseases and disorders analysis shows that the majority of genes with progressive promoter hyper‐ or hypomethylation are related to cancer. Molecular and cellular functions analysis reveals that differentially methylated loci are associated with cellular development, cellular growth and proliferation, and cell‐to‐cell signaling and interaction (Tables S2 and S3). Altogether, this analysis suggests that DNA methylation alterations have a functional impact on key pathways related to initiation and progression of PRCC.

### 3.4 A DNA methylation signature differentiating localized and advanced‐stage type 2 PRCC

**Figure 3:**
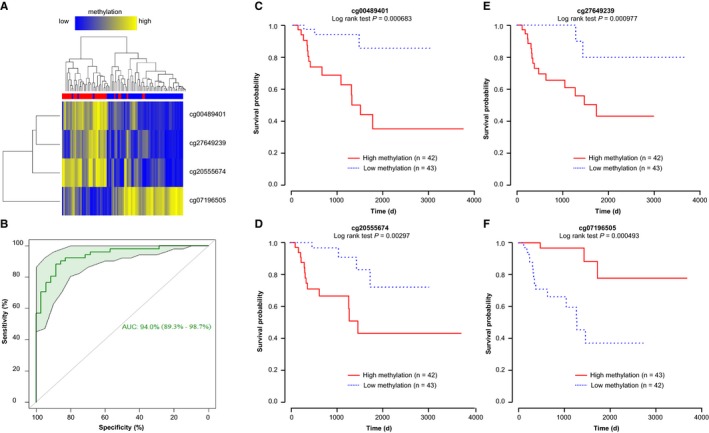
CpG markers that distinguish between localized and advanced‐stage type 2 PRCC. (A) Heatmap of methylation values for the four CpG markers (cg00489401, cg27649239, cg20555674, and cg07196505). Blue: localized‐stage type 2 PRCC samples; red: advanced‐stage type 2 PRCC samples. A color bar is shown, with low methylation in blue, and high methylation in yellow. (B) Area under the receiver operating characteristic curve (AUROC) analysis of the four CpG markers. The 95% confidence interval with 2000 stratified bootstrap replicates is shown in green. (C‐F) Kaplan‐Meier survival curves for methylation at cg00489401, cg27649239, cg20555674, and cg07196505, respectively. Significance based on log rank tests is shown below the CpG identifier. High methylation patients are shown in red, with low methylation in blue. The number of patients within each group is listed next to group labels for each AUROC plot

About 63 CpGs are identified as potential markers to discriminate between localized and advanced‐stage type 2 PRCC. Among them, five CpGs progressively gain methylation while one CpG progressively loses methylation from localized to advanced‐stage type 2 PRCC. However, two of the five progressively hypermethylated CpGs occur at single‐nucleotide polymorphism (SNP) sites, and thus were not considered further for analysis. Finally, three hypermethylated CpGs (cg00489401, cg27649239, cg20555674) and one hypomethylated CpG (cg07196505) were selected for further analysis. Hierarchical clustering demonstrated that 29 of 35 advanced‐stage type 2 PRCC clustered together and 48 of 51 localized‐stage type 2 PRCC clustered together (*P* < 0.0001) based on methylation at these four sites (Figure 3A). Moreover, a model that combines the selected four CpGs yields an area under the ROC curve (AUROC) value of 0.94, suggesting excellent sensitivity and specificity for differentiating between localized and advanced‐stage type 2 PRCC (Figure 3B). Survival analyses reveal that methylation levels of each of the selected four CpGs correlate significantly with patient survival (Figure 3C‐F, *P* < 0.05).

### 3.5 Copy number variation in localized and advanced‐stage type 2 PRCC

**Figure 4:**
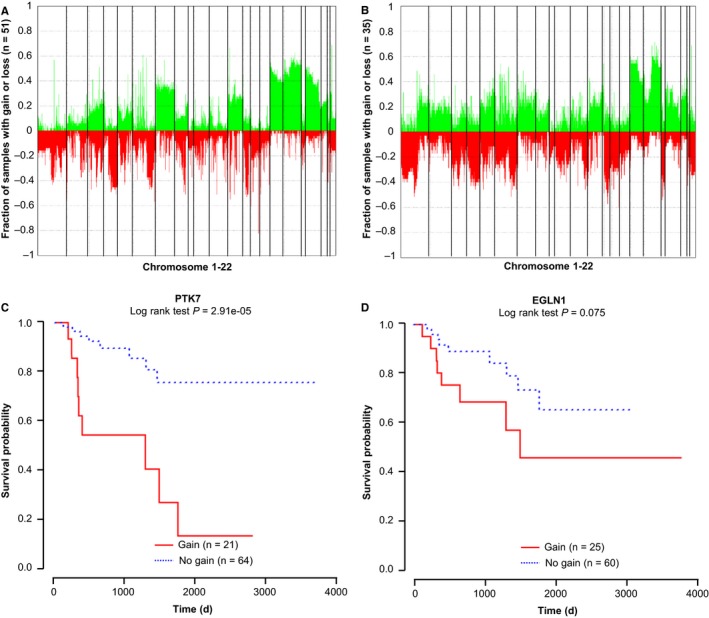
Copy number analysis in localized‐stage and advanced‐stage type 2 PRCC. (A‐B) Copy number variation in localized and advanced‐stage type 2 PRCC samples, relative to adjacent‐normal tissue. Gains are shown in green across chromosomes 1‐22 (separated by vertical bars), with losses in red for *PTK7* (A) and *EGLN1* (B). (C‐D) Kaplan‐Meier survival curves for CNV at *PTK7* and *EGLN1*. Significance based on log rank tests is shown below the CpG identifier. Patients with copy number gain are shown in red, copy number loss in denoted with blue. The number of patients within each group is listed next to group labels for each AUROC plot

Copy number analysis based on the raw 450k data, using adjacent‐normal tissue as control data, reveals differences between localized and advanced‐stage type 2 PRCC (Figure 4A,B, Table S4). Among the genes showing CNV, three (*PTK7*, *EGLN1*, *SMYD3*) have been reported to be related to RCC or cancer cell invasion.^14^ Gain of *PTK7* is observed in three of 51 cases (5.9%) of localized‐stage and 18 of 35 cases (51.4%) of advanced‐stage type 2 PRCC, and survival analysis demonstrates that *PTK7* gain is associated with poor survival (*P* < 0.0001). Gains at *EGLN1* trend toward statistical significance, but do not correlate with a significant influence on survival (*P* = 0.075) (Figure 4C,D). CNV at *SMYD3* has no effect on survival (*P* = 0.49, data not shown).

### 3.6 PTK7 is upregulated from localized to advanced‐stage type 2 PRCC

Compared with adjacent‐normal tissue, 5655 genes are upregulated and 3623 genes are downregulated in localized‐stage type 2 PRCC. From localized to advanced‐stage type 2 PRCC, 1831 genes are upregulated and 1317 genes are downregulated. Genes associated with the four previously identified differentially methylated CpGs (cg00489401, cg27649239, cg20555674, and cg07196505) are *FLT4*, *LBXCOR1*, *ARL5C*, and *A2BP1*, respectively. Only *FLT4* is downregulated in localized‐stage type 2 PRCC compared with adjacent‐normal samples. Expression of the other three genes shows no significant difference between adjacent‐normal samples and localized or advanced‐stage type 2 PRCC (not shown). Expression of *PTK7* is not significantly changed in localized‐stage type 2 PRCC when compared to adjacent‐normal samples. However, from localized to advanced‐stage type 2 PRCC, *PTK7* is significantly upregulated (logFC = 0.95, FDR = 0.02), suggesting that *PTK7* is associated with progression in type 2 PRCC. Moreover, while *PTK7* gain mostly occurs in advanced‐stage type 2 PRCC, samples with *PTK7* gain have higher gene expression than patients without gain in *PTK7* (log FC = 1.08, FDR = 0.01). Taken together, these findings reveal several potential genetic‐ and epigenetic‐based markers that could be developed into diagnostic tools to allow for better stratification of patients for clinical management.

## 4 DISCUSSION

The present study identifies a group of CpGs that progressively gain or lose DNA methylation from localized to advanced‐stage type 2 PRCC. Four CpGs (cg00489401, cg27649239, cg20555674, and cg07196505) differentiate between stages. Moreover, these four CpGs are associated with patient survival and may also serve as prognostic markers. Copy number analysis reveals that gain of *PTK7* occurs most frequently in advanced‐stage tumors and predicts poor survival. RNA‐seq analysis shows that *PTK7* is significantly upregulated in advanced stages, and correlates with copy number gain. Roles for *PTK7* in cancer cell invasion have been reported in several clinical and experimental studies,^15^, ^16^, ^17^ suggesting that *PTK7* represents a potential therapeutic target in advanced‐stage type 2 PRCC. Relationships between gene body DNA methylation and expression are complex and multifactorial, therefore whether the methylation changes we identified in four loci drive altered expression or splicing will require further investigation. These DMRs may, nonetheless, serve as useful markers of PRCC progression.

Our present study also demonstrates that genes targeted for progressive hypermethylation in the transition to advanced‐stage from localized PRCC are associated with adrenergic signaling, G protein signaling, and GABA receptor signaling. In the RCC cell line RCC7, acute activation of the β2‐adrenergic receptor‐regulated cancer cell migration occurs by promoting RhoA activation and influencing focal adhesion formation.^18^ G protein‐coupled receptors (GPCRs) play critical roles in the invasion and metastasis of cancer cells by activating Rho GTPases, and regulating cytoskeletal remodeling and angiogenesis. In the RCC cell line Caki‐2, GABA stimulation promotes cancer cell invasion via ERK1/2‐dependent upregulation of MMPs; an effect mediated mainly through the GABA‐B receptor.^19^ Thus, while DNA methylation changes may serve as prognostic markers in pRCC, they may also functionally influence the underlying biology of the disease.

Current recommendations according to the ESMO Clinical Practice Guidelines for RCC state that before treatment with ablative therapies, or in patients with metastatic disease prior to initiating systemic treatment, a renal biopsy should be performed to confirm malignancy.^20^ In elderly patients with significant comorbidities or those with a short‐life expectancy and solid renal tumors <4 cm, renal biopsy is also recommended for active surveillance.^20^ In addition to clinical and histopathological findings, molecular biomarkers may exert additional benefit for diagnosis and prognosis of type 2 PRCC. In the present study, we identify four DNA methylation markers that distinguish between localized and advanced‐stage type 2 PRCC with excellent sensitivity and specificity. Moreover, these four DNA methylation markers may be useful for predicting patient outcome. Because the sample size of type 2 PRCC from TCGA‐KIRP is small, we have not divided samples into training and validation sets, thus the differentially methylated sites we identified require validation as part of future studies. In addition, while the DNA methylation markers in our present study were identified from tumor tissue, several reports have demonstrated that blood‐ or urine‐based DNA methylation markers could be useful for noninvasive early diagnosis or prognosis of cancers such as prostate, bladder, colorectal, lung, and breast,^21^, ^22^ highlighting a logical next step for our studies with PRCC.

*PTK7* is an intrinsic component of the Wnt pathway and a member of the receptor tyrosine kinase family.^23^ In multiple cancers including esophageal squamous cell carcinoma, intrahepatic cholangiocarcinoma, breast cancer, and prostate cancer, *PTK7* is overexpressed and inversely correlated with overall survival.^24^, ^25^, ^26^ Copy number gain in *PTK7* is associated with increased gene expression, which is also observed in gastric cancer.^27^, ^28^ While located within the top 4% of all regions sustaining copy number gain in an independent lung cancer dataset, *PTK7* is also overexpressed in nonsmall cell lung cancers.^29^ In vitro experiments demonstrate that *PTK7* directs cancer cell motility and invasiveness, indicating that its amplification potentially plays a pro‐oncogenic role in driving tumorigenesis and/or metastasis.^24^, ^25^, ^26^, ^30^ By analyzing CNV and RNA‐seq data, our study shows that *PTK7* may serve a prognostic biomarker and a potential treatment target in advanced‐stage type 2 PRCC. A phase II study reported that sunitinib, a multi‐targeted receptor tyrosine kinase inhibitor, was effective in treating type 1 and 2 metastatic PRCC.^31^ As a receptor tyrosine kinase, *PTK7* is a predicted target of sunitinib.^32^ Therefore, it is possible that the effect of sunitinib in PRCC may be partly mediated through inhibition of *PTK7*. PF‐06647020, an antibody‐drug conjugate targeting *PTK7*, induces sustained tumor regression in patients with triple‐negative breast cancer, ovarian cancer, and nonsmall cell lung cancer.^33^ Whether PF‐06647020 has a similar effect in advanced‐stage type 2 PRCC remains to be investigated.

In conclusion, through analysis of 450K methylation data from TCGA‐KIRP, we identified four CpGs that may serve as DNA methylation markers for differentiating between localized to advanced‐stage type 2 PRCC. Moreover, these four CpGs may also be useful as prognostic markers. CNV analysis reveals that copy number gain in *PTK7* is associated with poor survival and higher expression from localized‐stage to advanced‐stage type 2 PRCC, suggesting that *PTK7* represents a promising treatment target for advanced‐stage type 2 PRCC.

## CONFLICT OF INTEREST

None to be declared.

## Data Availability

TCGA‐KIRP 450k array data was downloaded from the GDC (https://gdc-portal.nci.nih.gov/legacy-archive/search/f).
