# Supplementary material for: In silico DNA methylation analysis identifies potential prognostic biomarkers in type 2 papillary renal cell carcinoma
Source: Cancer Med. 2019 Jul 30;8(12):5760–8. doi: 10.1002/cam4.2402 (PMC6745825; doi:10.1002/cam4.2402)
Supplement: Supplementary file 2 [file CAM4-8-5760-s002.doc]

| **Supplemental Table S2. Genes showing progressive promoter hypermethylation from localized-stage to advanced-stage type 2 PRCC (IPA analysis).** | | |
| --- | --- | --- |
| **Top Canonical Pathways** |  |  |
| Name | p-value | Overlap |
| Adrenergic Signaling | 1.03E-04 | 13.8% (12/87) |
| Gi Signaling | 1.78E-04 | 11.7% (14/120) |
| GABA Receptor Signaling | 1.99E-04 | 14.9% (10/67) |
| cAMP-mediated signaling | 7.69E-04 | 8.6% (19/221) |
| Gs Signaling | 8.57E-04 | 11.0% (12/109) |
|  |  |  |
| **Top Diseases and Bio Functions** |  |  |
| **Diseases and Disorders** |  |  |
| Name | p-value | #Molecules |
| Cancer | 1.14E-03 - 1.28E-27 | 747 |
| Organismal Injury and Abnormalities | 1.22E-03 - 1.28E-27 | 755 |
| Gastrointestinal Disease | 9.89E-04 - 8.68E-23 | 665 |
| Dermatological Diseases and Conditions | 4.25E-04 - 2.96E-12 | 354 |
| Hepatic System Disease | 2.87E-06 - 2.72E-07 | 318 |
| **Molecular and Cellular Functions** |  |  |
| Name | p-value | #Molecules |
| Cellular Development | 1.29E-03 - 6.37E-16 | 281 |
| Cellular Growth and Proliferation | 1.29E-03 - 2.69E-12 | 316 |
| Cell Morphology | 9.93E-04 - 8.63E-11 | 230 |
| Cell-To-Cell Signaling and Interaction | 1.02E-03 - 3.05E-08 | 88 |
| Gene Expression | 2.07E-05 - 1.35E-07 | 163 |
