# Supplementary material for: In silico DNA methylation analysis identifies potential prognostic biomarkers in type 2 papillary renal cell carcinoma
Source: Cancer Med. 2019 Jul 30;8(12):5760–8. doi: 10.1002/cam4.2402 (PMC6745825; doi:10.1002/cam4.2402)
Supplement: Supplementary file 3 [file CAM4-8-5760-s003.doc]

| **Supplemental Table S3. Genes showing progressive promoter hypomethylation from localized-stage to advanced-stage type 2 PRCC (IPA analysis).** | | |
| --- | --- | --- |
| **Top Canonical Pathways** |  |  |
| Name | p-value | Overlap |
| Graft-versus-Host Disease Signaling | 6.99E-03 | 6.2% (3/48) |
| Differential Regulation of Cytokine Production in Macrophages and T Helper Cells by IL-17A and IL-17F | 9.24E-03 | 11.1% (2/18) |
| Inflammasome pathway | 1.25E-02 | 9.5% (2/21) |
| Gi Signaling | 1.67E-02 | 3.3% (4/120) |
| Hepatic Fibrosis / Hepatic Stellate Cell Activation | 1.71E-02 | 2.7% (5/183) |
|  |  |  |
| **Top Diseases and Bio Functions** |  |  |
| **Diseases and Disorders** |  |  |
| Name | p-value | #Molecules |
| Cancer | 1.62E-02 - 1.19E-20 | 161 |
| Dermatological Diseases and Conditions | 1.62E-02 - 1.19E-20 | 114 |
| Organismal Injury and Abnormalities | 1.62E-02 - 1.19E-20 | 162 |
| Endocrine System Disorders | 1.62E-02 - 1.17E-07 | 72 |
| Gastrointestinal Disease | 1.62E-02 - 1.17E-07 | 143 |
| **Molecular and Cellular Functions** |  |  |
| Name | p-value | #Molecules |
| Cell-To-Cell Signaling and Interaction | 1.62E-02 - 6.58E-05 | 61 |
| Cellular Development | 1.62E-02 - 6.58E-05 | 18 |
| Cellular Growth and Proliferation | 1.62E-02 - 6.58E-05 | 21 |
| Gene Expression | 1.62E-02 - 6.58E-05 | 4 |
| Lipid Metabolism | 1.62E-02 - 6.58E-05 | 6 |
