# Supplementary material for: In silico DNA methylation analysis identifies potential prognostic biomarkers in type 2 papillary renal cell carcinoma
Source: Cancer Med. 2019 Jul 30;8(12):5760–8. doi: 10.1002/cam4.2402 (PMC6745825; doi:10.1002/cam4.2402)
Supplement: Supplementary file 4 [file CAM4-8-5760-s004.docx]

| **Supplemental Table S4. Genes with copy number variation in type 2 PRCC.** | | | | | |
| --- | --- | --- | --- | --- | --- |
| Gene | Gain | | Gene | Loss | |
|  | Advanced-stage (n=35) | Localized-stage (n=51) |  | Advanced-stage (n=35) | Localized-stage (n=51) |
| *PTK7* | 18 (51.43%) | 3 (5.88%) | *SMYD3* | 8 (22.86%) | 27 (52.94%) |
| *FLJ20397* | 14 (40%) | 6 (11.76%) | *FLJ25715* | 7 (20%) | 2 (3.92%) |
| *C20orf121* | 11 (31.43%) | 4 (7.84%) | *CTDP1* | 7 (20%) | 2 (3.92%) |
| *TSPAN32* | 9 (25.71%) | 1 (1.96%) | *LOC645411* | 7 (20%) | 2 (3.92%) |
| *LOC644125* | 8 (22.86%) | 29 (56.86%) | *KCNG2* | 7 (20%) | 2 (3.92%) |
| *EGLN1* | 8 (22.86%) | 1 (1.96) | *PQLC1* | 7 (20%) | 2 (3.92%) |
| *ASH1L* | 8 (22.86%) | 3 (5.88%) | *LOC440498* | 7 (20%) | 2 (3.92%) |
|  |  |  | *TXNL4A* | 7 (20%) | 2 (3.92%) |
|  |  |  | *MBP* | 7 (20%) | 2 (3.92%) |
|  |  |  | *VPS13B* | 5 (14.29%) | 16 (31.37%) |
